# The MexJK Multidrug Efflux Pump Is Not Involved in Acquired or Intrinsic Antibiotic Resistance in *Pseudomonas aeruginosa*, but Modulates the Bacterial Quorum Sensing Response

**DOI:** 10.3390/ijms23147492

**Published:** 2022-07-06

**Authors:** Rafael Amieva, Teresa Gil-Gil, José Luis Martínez, Manuel Alcalde-Rico

**Affiliations:** 1 Centro Nacional de Biotecnología, CSIC, Darwin 3, 28049 Madrid, Spain; raamieva@ucm.es (R.A.); tgil@cnb.csic.es (T.G.-G.); 2 SALUVET Group, Animal Health Department, Complutense University of Madrid, Ciudad Universitaria s/n, 28040 Madrid, Spain; 3 Programa de Doctorado en Biociencias Moleculares, Universidad Autónoma de Madrid, 28049 Madrid, Spain; 4 Grupo de Resistencia Antimicrobiana en Bacterias Patógenas y Ambientales (GRABPA), Instituto de Biología, Pontificia Universidad Católica de Valparaíso, Valparaíso 2373223, Chile; 5 Millennium Initiative for Collaborative Research on Bacterial Resistance (MICROB-R), Santiago 7550000, Chile

**Keywords:** *Pseudomonas aeruginosa*, quorum sensing, antibiotic resistance, PQS, pyoverdine, MexJK

## Abstract

Multidrug efflux pumps are critical elements in both intrinsic and acquired antibiotic resistance of bacterial populations. Consequently, most studies regarding these protein machineries focus on this specific phenotype. Nevertheless, different works show that efflux pumps participate in other aspects of bacterial physiology too. Herein, we study the *Pseudomonas aeruginosa* multidrug efflux pump MexJK. Previous studies, using model strains lacking MexAB-OprM and MexCD-OprJ efflux pumps, support that MexJK can extrude erythromycin, tetracycline, and triclosan. However, the results here reported indicate that this potential increased extrusion, in a mutant overexpressing *mexJK*, does not alter the antibiotics susceptibility in a wild-type genetic background where all intrinsic multidrug efflux pumps remain functional. Nevertheless, a clear impact on the quorum sensing (QS) response, mainly in the Pqs-dependent QS regulation network and in the expression of Pqs-regulated virulence factors, was observed linked to *mexJK* overexpression. The production of the siderophore pyoverdine strongly depended on the level of *mexJK* expression, suggesting that MexJK might participate in *P. aeruginosa* pyoverdine-dependent iron homeostasis. All in all, the results presented in the current article support that the functions of multidrug efflux pumps, as MexJK, go beyond antibiotic resistance and can modulate other relevant aspects of bacterial physiology.

## 1. Introduction

*Pseudomonas aeruginosa* is a prevalent nosocomial pathogen, as well as an important causative agent of chronic lung infections in patients with cystic fibrosis and chronic obstructive pulmonary diseases [1,2,3]. The success of *P. aeruginosa* as an opportunistic pathogen is due to the great adaptability of this bacterium to colonize different habitats, including a variety of hosts, and to the presence in its genome of a large number of genes encoding virulence factors [4,5]. In addition, *P. aeruginosa* presents a characteristic low susceptibility to several antibiotics and can easily acquire high-level antibiotic resistance [6,7,8]. All these factors place *P. aeruginosa* as one of the priority pathogens against which the development of new antibiotics is critical [9,10].

Resistance-nodulation-cell division (RND) efflux pumps are fundamental contributors to bacterial antibiotic resistance. Their expression is finely controlled by one or more regulatory elements [11,12,13,14], most frequently repressors. Since the expression of these efflux pumps is usually low, just some of them contribute to intrinsic resistance [13,14]. However, when overexpressed, efflux pumps can contribute to phenotypic resistance by transient induction of their expression, which is triggered by effectors or by growing conditions [14,15], and to acquired resistance via mutations in both RND efflux pump subunits and the elements that regulate their expression [14,16,17].

Since multidrug resistance (MDR) efflux pumps are ancient, very well conserved protein machineries present in all organisms, it has been suggested that they have functions in bacterial physiology that go beyond antibiotic resistance [18,19,20]. These processes include the response to stress situations [21] or to host defenses [22], or the modulation of the quorum sensing (QS) signaling [23,24,25,26], among others. The QS response is based on a cell-to-cell communication system that modifies the behavior of a bacterial population in response to changes in population density [27,28]. This mechanism is based on the synthesis, release, and detection of autoinducer compounds, known as QS signal molecules (QSSMs) [28,29]. The progressive accumulation of these QSSMs promotes the expression of autoinducers’ biosynthetic pathways, thus giving a positive feedback to the system [30]. When QSSMs concentrations overcome a threshold, the QS response is activated, regulating the expression of a wide number of QS-regulated genes, including, in the case of *P. aeruginosa*, those involved in the production of virulence factors [31,32,33,34,35], thus producing a coordinated population response [36,37]. The QS regulatory network of *P. aeruginosa* [27,38] is based on the production of two different types of QSSMs: the *N*-acyl-L-homoserine lactones (AHLs) and the 2-alkyl-4(1H)-quinolones (AQs) [27,39]. These molecules are synthesized and detected by the *Las*, *Rhl*, and *Pqs* systems. The *Las* and *Rhl* systems depend on the LasI and RhlI synthases that produce the molecules *N*-(3-oxododecanoyl)-L-homoserine lactone (3-oxo-C12-HSL) and *N*-butanoyl-L-homoserine lactone (C4-HSL), respectively, whose expression is positively regulated by the LasR and RhlR transcriptional factors that detect the presence of their mentioned specific autoinducers. The third system, *Pqs*, is mainly based on the synthesis of the *Pseudomonas* quinolone signal (PQS) and its most immediate precursor, 2-heptyl-4-hydroxyquinoline (HHQ), driven by its biosynthetic pathway encoded by the operon *pqsABCDE* and *pqsH*. The expression of this pathway is positively regulated by PqsR, which is activated by the binding of the mentioned QSSMs. It is generally assumed that these three QS systems are related to each other in a hierarchized way, with the *Las* system controlling the activity of the *Rhl* and *Pqs* systems, which then modulate their own activity and other QS systems [40,41]. However, there are other elements, such as PqsE, and global regulators, such as VqsM or GacA/GacS, that function as QS modulators, allowing *P. aeruginosa* to adapt its physiology to specific environmental conditions [42,43,44,45,46].

**Figure 1:**
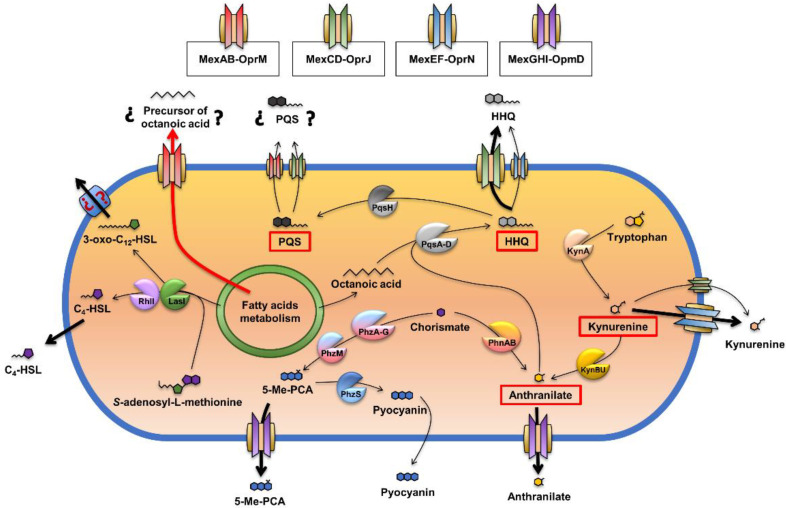
Schematic representation of the synthesis of QSSMs and their relationship with RND efflux activity in *P. aeruginosa*. Previous studies have shown that MexAB-OprM, MexCD-OprJ, and MexEF-OprN are efflux system able to extrude QS-related compounds, a feature that produces an impaired QS response and virulence factors production. Therefore, the overexpression of MexAB-OprM has been associated with an impaired production (not extrusion) of one of the immediate precursors of HHQ, octanoic acid, rather than with a nonphysiological extrusion of 3-oxo-C12-HSL as was initially stated [24,48]. In our work, we suggested that this lower availability of octanoic acid is probably due to the efflux of some of the intermediates in fatty acid metabolism that function as precursors of octanoate synthesis. With respect to MexCD-OprJ and MexEF-OprN, both of them are able to efflux HHQ and the AQs precursor kynurenine [23,25,49], but with different efficiency, being the extrusion of either HHQ or kynurenine most relevant in mutants that overexpress MexCD-OprJ or MexEF-OprN, respectively. Some other studies have also associated the activity of the MexGHI-OpmD efflux system with the QS network at two different levels: (i) extruding anthranilate, which is the other one immediate precursor of HHQ [26], and (ii) extruding 5-Me-PCA, the immediate precursor of pyocyanin, a QS-controlled virulence factor that in turns drives the expression of some other QS-related genes [50]. Altogether, these results support that RND efflux systems are a key element in the modulation of the QS response at different levels [19,20].

Different studies [23,24,26,47,48,49] have evidenced that several RND efflux systems are implicated in the *P. aeruginosa* QS response (Figure 1). For example, overexpression of MexAB-OprM leads a reduction in AQs production, due to a decrease in the availability of the precursor octanoic acid [24]. Moreover, MexEF-OprN and MexCD-OprJ are able to efflux HHQ and kynurenine, both precursors of the PQS autoinducer, which leads to a defective QS response in those mutants that overexpress these systems [23,25]. Concerning MexGHI-OpmD, a QS-regulated efflux pump, it has been shown that it is able to extrude anthranilate, the immediate precursor of HHQ, and 5-methylphenazine-1-carboxylate (5-Me-PCA), which is the immediate precursor of the QS-controlled virulence factor, pyocyanin; hence, defects in *mexGHI-opmD* expression also impair the QS response [26,50]. Altogether, these results led us to propose that RND efflux systems could play an important role in the modulation of *P. aeruginosa* QS response through changes in the production or the extrusion of different QSSMs and/or their precursors.

The sequence of the *P. aeruginosa* PAO1 genome, and further analysis of several different isolates, showed that this bacterial species encodes in its core genome twelve intrinsic RND transporters, with MexAB-OprM [51,52], MexCD-OprJ [53,54], MexEF-OprN [55], and MexXY [56,57] being the ones with the greatest clinical relevance. However, the implication of other efflux pumps—as MexJK, here studied—on antibiotic resistance has been suggested based on in vitro assays, but it has not been analyzed in detail. MexJK is an efflux pump encoded by the *mexJK* operon, whose expression is proposed to be negatively regulated by a TetR family repressor, known as MexL [58,59,60]. In addition, different transcriptomics studies support that *mexJK* expression is under the control of the QS system [61,62,63,64], suggesting a link between this efflux pump and the *P. aeruginosa* QS response.

Studies on the role of this efflux pump in *P. aeruginosa* resistance have been performed just in antibiotic hypersusceptible strains, modified in the laboratory, which lack the most relevant RND efflux pumps contributing to *P. aeruginosa* intrinsic resistance [58,59,60]. Therefore, the role on antibiotic resistance that MexJK might have in a wild-type genomic background, in which the other RND efflux pumps are functional—the common isolates from patients—remains to be established.

Hence, in this work, we focus on the potential role that MexJK could have in both antibiotic resistance and modulation of the QS response in a wild-type genomic context. To reach this goal, two deletion mutants, *ΔmexL* and *ΔmexK*, which overexpress MexJK and produce a defective efflux system, respectively, were generated in the wild-type PAO1 strain. Our results showed that, while neither the loss of MexJK function nor *mexJK* overexpression produces significant changes in antibiotic susceptibility with respect to their parental *P. aeruginosa* PAO1 strain, the overproduction of this efflux system leads to impaired QS response and virulence factor production mainly due to a lower production of PQS and HHQ.

## 2. Results and Discussion

### 2.1. The Expression of MexJK Is Regulated by Both MexL and Growth Phase

It has been previously stated that *mexJK* expression is regulated by the repressor MexL [60]. Nevertheless, while the performed in vitro biochemical analyses were robust, *mexL* expression was measured in an indirect way, by using plasmids carrying reporters of its expression [59,60]; the level of expression of the native, chromosomally encoded *mexJK* was not determined in these studies. Further, the analysis of *P. aeruginosa* clinical isolates showed that the *mexK* level of expression does not always correlate with clear changes in MexL [65], casting some doubts on the mechanisms of regulation of the expression of this efflux pump. In addition, all studies regarding the role of MexJK have been performed in a *P. aeruginosa* strain lacking the multidrug efflux pumps *mexAB-oprM*—the main efflux pump contributing to *P. aeruginosa* intrinsic resistance—and *mexCD-oprJ* [58,59]. To ascertain the role of MexJK in *P. aeruginosa* antibiotic resistance and in other aspects of bacterial physiology, as well as to confirm the potential contribution of MexL in such processes, the effect of *mexJK* expression level changes must be studied in a wild-type genetic background.

**Figure 2:**
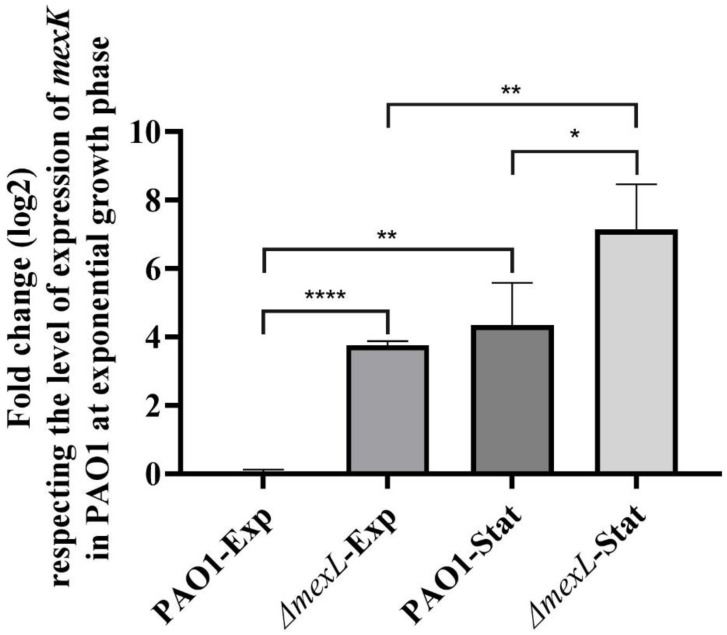
Effect of MexL and growth phase on *mexJK* expression. The expression of *mexK* was determined by RT-qPCR in both PAO1 and *ΔmexL* strains at stationary and exponential phases of growth. Fold change regarding the level of expression of *mexK* in PAO1 at exponential growth phase is presented. As shown, lack of MexL highly increased *mexK* expression, indicating that, in agreement with previous reports [59,60], MexL is a negative regulator of *mexJK*. In addition, expression was higher at stationary growth phase in both strains, indicating that growth-phase regulation of *mexK* expression is independent of MexL. Values that are significantly different by an unpaired two-tail *t*-test are indicated by asterisks as follows: * *p* < 0.05; ** *p* < 0.01; **** *p* < 0.0001.

For such purpose, a *ΔmexL* mutant and a *ΔmexK* mutant were obtained by homologous recombination in the wild-type *P. aeruginosa* PAO1 strain, as described in Methods. Once the *mexL*-deficient mutant was obtained, the expression of *mexK*, the gene encoding the RND subunit of the MexJK efflux system, in both exponential (OD_600_ = 0.6) and early stationary phases of growth (OD_600_ = 2.5), was analyzed by quantitative reverse transcription PCR (RT-qPCR) as described in Methods. Consistent with previous information [59,60], *mexK* was overexpressed in the mutant lacking *mexL* as compared with the wild-type strain (Figure 2), supporting that MexL is a negative regulator of *mexJK* expression. In addition, the comparison of *mexK* expression between the stationary and exponential phases in PAO1 and *ΔmexL* mutant showed that this efflux system is similarly induced during the stationary growth in both strains (Figure 2), evidencing that MexL does not play a key role in the growth phase-dependent expression of MexJK. The increase in *mexK* expression observed in stationary growth phase is in agreement with reports indicating that *mexJK* expression could be under the control of the QS system, [61,62,63,64,66] and that this control is observed just in stationary phase, but not in exponential phase [62]. Our results, together with previous findings, suggest that *mexJK* expression involves different layers of regulation that should include some other regulators in addition to the local repressor MexL.

### 2.2. Overexpression of MexJK Efflux System Does Not Change P. aeruginosa Antibiotics’ Susceptibility Profile

It has been described that MexJK is able to extrude erythromycin and tetracycline when this pump is associated with the outer membrane protein (OMP) OprM, while it can extrude triclosan when it is associated with OpmH, another OMP that has been previously shown to be implicated in triclosan resistance in combination with the TriABC efflux system [58,59,67]. However, as stated above, this antibiotics substrate profile of MexJK has been determined using an hypersusceptible strain generated in the laboratory and lacking two of the main MDR efflux pumps involved in the acquisition of antibiotic resistance by clinical *P. aeruginosa* isolates: MexAB-OprM and MexCD-OprJ. This means that we still ignore the contribution of the expression of this efflux pump to the antibiotic resistance phenotype of wild-type strains, harboring the whole set of *P. aeruginosa* MDR efflux pumps, including the most clinically relevant: MexAB-OprM [51,52], MexCD-OprJ [53,54], MexEF-OprN [55], and MexXY [56,57]. Consequently, although mutants overexpressing MexJK have been reported in clinics [65,68], the actual role of this efflux pump in the acquisition of antibiotic resistance by clinical *P. aeruginosa* isolates is still unknown.

The minimum inhibitory concentration (MIC) of a wide variety of antibiotics belonging to different families was determined in the wild-type PAO1 strain and its derivatives *ΔmexL* and *ΔmexK* by using MIC Test Strips. The results obtained (Table 1) show that, in this genetic wild-type background in which the intrinsic RND efflux systems implicated in antibiotic resistance are present, both the overexpression and loss of function of the MexJK efflux system have a minor impact on antibiotic susceptibility. From these results, we can conclude that, in a wild-type context, MexJK is not involved in intrinsic resistance to antibiotics and that its overexpression has no effect on *P. aeruginosa’s* acquired resistance to antimicrobials.

**Figure 3:**
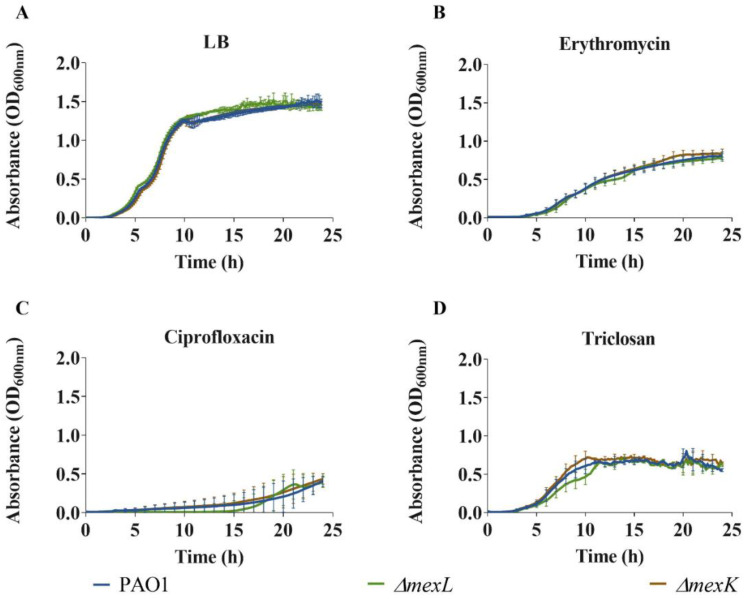
Analysis of growth in LB for PAO1, *ΔmexL*, and *ΔmexK* strains in (**A**) absence and in (**B**–**D**) presence of antimicrobials. Growth (OD_600_) was measured for 24 h in the presence of compounds suggested to be MexJK substrates—(**B**) erythromycin and (**C**) triclosan—as well as in the presence of one antibiotic that has not been proved to be related to MexJK—(**D**) ciprofloxacin. The concentrations of the antimicrobials used were 64 μg/mL erythromycin, 0.06 μg/mL ciprofloxacin, and 109 μg/mL triclosan. Error bars indicate standard deviations for the results from three independent replicates. As shown, neither the absence nor the overexpression of *mexJK* produced any relevant effect on *P. aeruginosa* growth under the tested conditions. These data indicate that *mexJK* overexpression or the lack of this efflux pump does not produce a relevant effect on *P. aeruginosa* fitness and reinforce the idea that *mexJK* does not contribute to erythromycin, ciprofloxacin, or triclosan resistance in a wild-type genomic context.

To further confirm the lack of effect in antibiotic susceptibility of MexJK in a wild-type background, the wild-type strain and its derived *ΔmexL* and *ΔmexK* mutants were grown in absence and in presence of the antimicrobials previously suggested to be MexJK substrates, namely, erythromycin and triclosan, using ciprofloxacin as a control of antibiotics not reported to be extruded by MexJK. As shown in Figure 3, the growth of the three strains was the same in all tested conditions, further supporting that MexJK does not contribute to intrinsic or to acquired resistance to antimicrobials of wild-type *P. aeruginosa*.

### 2.3. The Overexpression of the MexJK Efflux System Modifies the Expression of Several Genes Related to the P. aeruginosa QS Response

We have determined that *mexJK* increased expression does not contribute to antibiotic resistance in a wild-type genomic background. However, clinical isolates overexpressing this efflux pump have been reported [65,68]. This fact, together with the simultaneous selection of mutations in genes encoding QS regulators and in *mexL* that has been reported in *P. aeruginosa* biofilms [69], suggests that a main role of this efflux system could be more related to adaption to in-host growing conditions, virulence, or QS response rather than to antibiotic resistance. In favor of this hypothesis is the finding that the disruption of the QS response through mutations in *pqsA* (implicated in AQs synthesis), *rhlR*, or *lasR* (two master regulators of QS) renders the downregulation of *mexJK* expression [61,66]. It has been reported that this QS-dependent regulation of *mexJK* expression takes place just at the stationary phase of growth, since neither the overexpression of LasR, RhlR, or RpoS, nor the exogenous addition of AHLs is able to change the expression of this efflux system in the exponential growth phase [62]. Moreover, a hyperinduction of *pqsE* (a noncanonical QS modulator with dual role in activation/repression of AQs synthesis) is associated with the repressed expression of *pqsABCDE* and *mexJK* operons [66]. These results further support the association between MexJK and the QS response, suggesting that this efflux system is positively regulated along the stationary phase by AHLs and AQs but it is also repressed when PqsE is highly expressed, a situation that is more associated to late stationary growth [70].

Although available transcriptomic information supports that *mexJK* expression is regulated by QS, the possibility that this efflux system may modulate both QS response and *P. aeruginosa* virulence remains unexplored. To address this issue, we measured the expression, in both exponential and early stationary growth phases, of a set of genes involved in QS signaling, either because of their role in the production of QSSMs or because their expression is controlled by *P. aeruginosa* QS response and they participate in the production of different QS-regulated virulence factors. Since the QS response depends on the bacterial growth phase, and the overexpression of efflux pumps might produce an effect on bacterial fitness, we first compared the growth kinetics of the strains used in this work. As shown in Figure 3, neither the mutant lacking *mexK* nor the one overexpressing the MexJK efflux pump presented significant differences in their growth with respect their parental strain, PAO1.

**Figure 4:**
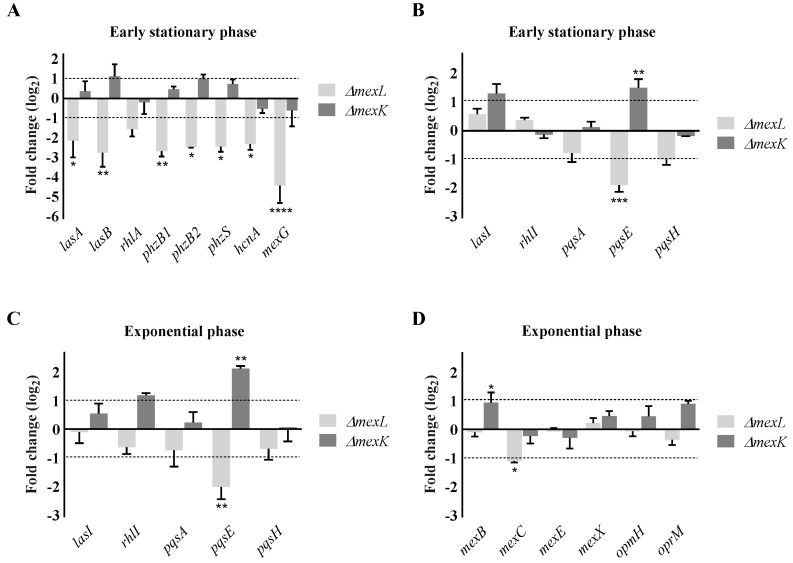
Overexpression of the MexJK efflux system affects the expression levels of QS signaling genes. Total RNA was extracted for PAO1, *ΔmexL*, and *ΔmexK* at (**A**,**B**) early stationary (OD_600_ = 2.5) and (**C**,**D**) exponential phases of growth (OD_600_ = 0.6). The expression was determined by RT-qPCR for (**A**) genes regulated by QS, (**B**,**C**) genes responsible for QSSMs production, and (**D**) genes belonging to various RND pumping systems. The figure represents the fold change of the expression of these genes in the *ΔmexL* or *ΔmexK* strains, with respect to the one observed in PAO1. Values that are significantly different by an unpaired two-tail *t*-test are indicated by asterisks as follows: * *p* < 0.05; ** *p* < 0.01; *** *p* < 0.001; **** *p* < 0.0001.

Once this control was established, we firstly measured the expression of genes involved in the production of elastases (*lasA* and *lasB*), rhamnolipids (*rhlA*), phenazines (*phzB1*, *phzB2*, *phzS*, and *mexG*), and cyanide (*hcnA*); all of them controlled by the QS regulation network in early stationary phase of growth. As shown in Figure 4A, the expression of all of these genes, excluding *rhlA*, was significatively lower in the mutant that overproduces MexJK, while the *mexK* defective mutant presented similar or slightly higher (no significant) expression compared with the wild-type strain. Further, the expression of genes involved in the production of QSSMs both in early stationary phase and in exponential phase was analyzed (Figure 4B,C). The results showed that, despite there being slight changes in the expression of the genes involved in the production of 3-oxo-C12-HSL (*lasI*), C4-HSL (*rhlI*), and PQS/HHQ (*pqsA*/*pqsH*) in *ΔmexK* or *ΔmexL* during the exponential and early stationary phases of growth, these changes were not significant with respect to the PAO1 wild-type strain. However, the expression levels of *pqsE*, a key component of QS regulation mediated by PQS and HHQ, decreased when *mexJK* was constitutively expressed (*ΔmexL* strain) and increased when the MexJK efflux system was defective (*ΔmexK* strain) in the two analyzed growth phases. These results suggest that MexJK activity could be mainly affecting the *Pqs*-dependent regulation of QS, and more precisely, the regulation network that is under the control of PqsE. Finally, in order to know if some other QS-related efflux pumps or their associated OMPs could be implicated in the observed phenotypes, we measured the expression of *mexB*, *mexC*, *mexE*, *mexX*, *opmH*, and *oprM* in the exponential phase of growth (Figure 4D). The results showed a small imbalance in the expression of *mexB* and *mexC* in the *ΔmexK* and *ΔmexL* strains, respectively. However, this small change in the level of expression of the studied efflux pumps was unlikely enough to justify the phenotype observed in these two strains. Taking into account that MexAB-OprM and MexCD-OprJ may have a role in the modulation of the QS response, it is not surprising that their expression could be affected by other RND efflux systems with potential roles in QS, thus fine tuning their effects over this cell-to-cell communication network.

### 2.4. The Overexpression of the MexJK Efflux System Is Associated with a Decreased Production of PQS and HHQ Autoinducer Compounds

Following our observation that the mutant overexpressing MexJK has a decreased expression of relevant genes belonging to the QS regulon, we wanted to know if, similarly to other RND efflux systems, a decreased production of QSSMs could be the underlying cause of this transcriptional imbalance. Firstly, using a TLC-based assay, we analyzed the level of 3-oxo-C12-HSL, C4-HSL, PQS, and HHQ accumulated by PAO1, *ΔmexL*, and *ΔmexK* cultures grown up to late exponential (AHLs) or early stationary phase (AQs) as described in methods [24].

**Figure 5:**
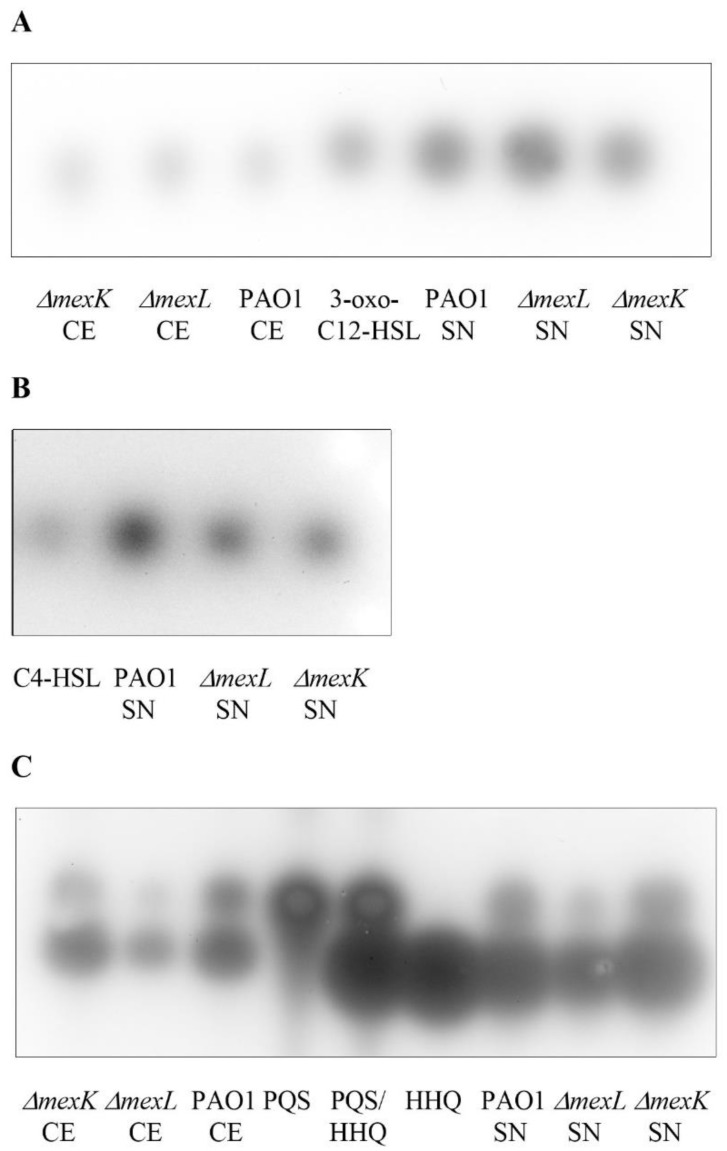
Accumulation of QSSMs both in supernatant and in cellular extracts of PAO1, *ΔmexL*, and *ΔmexK* strains grown in LB medium. Thin-layer chromatography analysis of both cell extracts (CEs) and supernatants (SNs) of PAO1, *ΔmexL*, and *ΔmexK* cultures grown to late exponential (AHLs; OD_600_ = 1.7) or early stationary phase (AQs; OD_600_ = 2.5) coupled to the growth of (**A**) LasR, (**B**) RhlR, or (**C**) PqsR-based biosensor strain. Since C4-HSL freely diffuses through the plasma membrane and hence should reach an equilibrium between the extracellular and intracellular levels [48], only supernatants were measured. No differences in 3-oxo-C12-HSL accumulation between the strains were observed. Although a decrease in C4-HSL accumulation was observed, this was found in both *ΔmexL* and *ΔmexK* strains, evidencing that MexJK has a minor impact on this phenotype. The lower PQS and HHQ accumulation in SN and CE of *ΔmexL* with respect to PAO1 and *ΔmexK*, was the most evident change associated with MexJK overproduction.

As Figure 5 shows, there were no changes in the accumulation of 3-oxo-C12-HSL among the three strains, neither in cellular extracts (CE) nor in supernatants (SN). In the case of C4-HSL, the wild-type strain presented a slightly higher accumulation than did the *ΔmexL* mutant in supernatant extracts; nevertheless, similar differences were observed in the *ΔmexK* mutant, thus indicating that the overexpression of MexJK has a minor impact in C4-HSL production (cellular extracts were not analyzed because C4-HSL freely diffuses across the plasma membrane [48]). However, a clear decrease in the accumulation of PQS and HHQ was observed in *ΔmexL* cultures with respect to those in PAO1 and *ΔmexK* (Figure 5).

**Figure 6:**
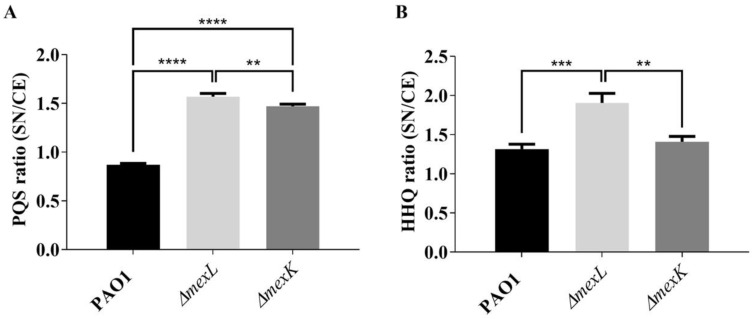
Determination of the ratio supernatant/cell extract (SN/CE) of PQS and HHQ. The TLC-spots corresponding to (**A**) PQS and (**B**) HHQ were quantified by densitometry using the ImageJ software, and the ratio between the HHQ and PQS present in the supernatant with respect to cell extract was calculated. The results showed that, although *ΔmexL* presented a higher PQS and HHQ ratio with respect to PAO1, only the HHQ ratio could be interpreted as a consequence of efflux pump overexpression. Values that are significantly different by an unpaired two-tail *t*-test are indicated by asterisks as follows: ** *p* < 0.01; *** *p* < 0.001; **** *p* < 0.0001.

Despite the fact that this lower AQs accumulation was found in both supernatant (PQS) and cellular extracts (PQS and HHQ), a higher HHQ ratio (SN accumulation vs. CE accumulation) in *ΔmexL* cultures with respect to PAO1 and *ΔmexK* was observed (Figure 6A,B). This last result suggests that, similarly to what has been described for other RND efflux systems [24,25], MexJK could be able to extrude HHQ. Overall, these results support that the synthesis and accumulation of these autoinducer compounds is associated with MexJK activity.

**Figure 7:**
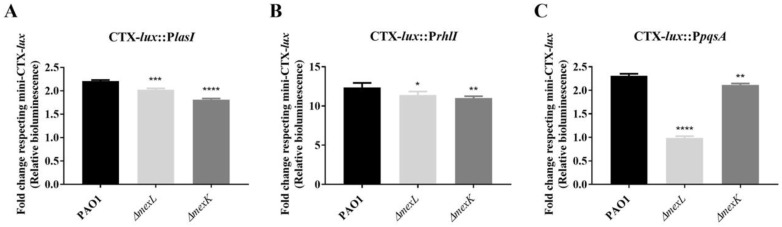
Analysis of the activation of promoters involved in QSSMs synthesis. Time-course of (**A**) P*lasI*, (**B**) P*rhlI*, and (**C**) P*pqsA* expression was analyzed in PAO1, *ΔmexL*, and *ΔmexK* strains growing in LB medium for 20 h, using a chromosomal insertion of the reporter construction of each promoter followed by the operon *luxCDABE*. The total area under the curve was quantified and is represented. As shown, luminesce driven by the *pqsA* promoter was strongly impaired in the strain that overproduces the MexJK efflux system. Values that are significantly different with respect to those obtained in the PAO1 strain by an unpaired two-tail *t*-test are indicated by asterisks as follows: * *p* < 0.05; ** *p* < 0.01; *** *p* < 0.001; **** *p* < 0.0001.

In order to explore this hypothesis in depth, we analyzed the activation of the different promoters that control the expression of the genes involved in QSSMs synthesis (P*lasI*, P*rhlI*, and P*pqsA*) throughout the growth of PAO1, *ΔmexL*, and *ΔmexK* cultures. For this purpose, a site-specific insertion in the chromosome of each strain with *luxCDABE*-based constructions, in which the bioluminescence emitted is under the control of one of the three QS promoters of interest, was carried out as indicated in Methods [71,72]. Once the new strains were obtained (Table 2), the absorbance and bioluminescence emitted were monitored for 20 h in a plate-reader machine. The ratio between bioluminescence (RLU) and absorbance (OD_600_) for each strain and time was calculated and normalized to the corresponding control strain as described in Methods. The results (Figure 7) showed a similar behavior to that observed by TLC-based assays, since the activation of the P*pqsA* promoter was the most impaired by the overproduction of the MexJK efflux system. Notwithstanding, the *ΔmexL* strain presented a small decrease in P*lasI* and P*rhlI* activation, but the MexJK defective mutant also presented differences in the same direction. This last fact proves that MexJK activity is not responsible for these observations. Altogether, our results showed that the overexpression of the MexJK efflux system negatively impacts the production of PQS and HHQ and leads to a defective *Pqs*-dependent response. Similar results have been reported when other RND efflux systems are overexpressed in *P. aeruginosa*. However, the underlying cause of this impairment in AQs synthesis was different in all cases, since the mutants overexpressing MexAB-OprM, MexCD-OprJ, and MexEF-OprN were mainly associated with an impairment in octanoate production (immediate precursor of HHQ), HHQ extrusion, and kynurenine (AQs precursor) extrusion, respectively (Figure 1). Although the underlying cause of the impaired AQs production in the mutant overexpressing MexJK remains unclear, our results support that HHQ extrusion could be implicated (Figure 7). Moreover, the fact that *mexB*, *mexC*, and *mexE* are not overexpressed (Figure 4D) in the MexJK-overexpressing mutant (*ΔmexL* strain) supports that MexJK could have a specific role in the regulation of QS response, even in a PAO1 genetic background with the main RND efflux system available. This new evidence supports that RND efflux systems may function as modulators of the QS response due to their “apparent redundancy” and through the differential expression of several RND systems such as MexAB-OprM, MexCD-OprJ, MexEF-OprN, MexGHI-OpmD, and MexJK [20,23,24,25,26,47,48,50], which probably help *P. aeruginosa* to adapt its population-scale response to the needs of the environment (Figure 1).

### 2.5. Differential Activity of MexJK Efflux System Alters the Production of QS-Regulated Virulence Factors

Once we established that changes in the levels of *mexJK* expression modify the expression of QS-regulated genes and the production of QS autoinducers, we aimed to validate these findings at a phenotypic level. To test if the overexpression of MexJK, similarly to the overexpression of other RND efflux systems in *P. aeruginosa*, has an effect on the production of QS-regulated virulence factors, we analyzed the production of pyocyanin, pyoverdine, elastase, rhamnolipids, and biofilm, as well as the swarming and swimming motility in PAO1, *ΔmexL*, and *ΔmexK* strains.

**Figure 8:**
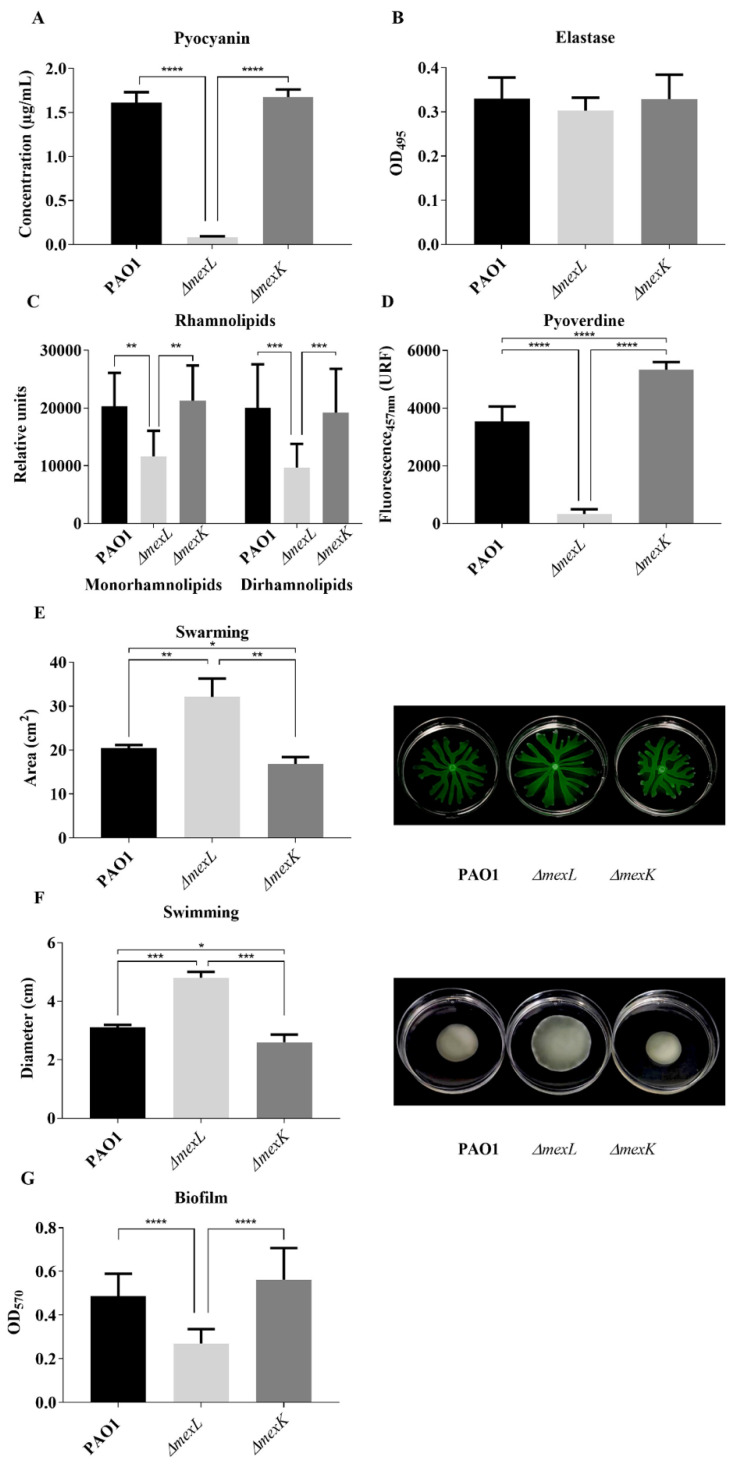
Analysis of the overexpression and deletion of the MexJK efflux pump in the production of different virulence factors regulated by QS. The production of (**A**) pyocyanin, (**B**) elastase, (**C**) rhamnolipids, and (**D**) pyoverdine was analyzed in supernatants of PAO1, *ΔmexL*, and *ΔmexK* strains grown in LB medium for 20 h. For (**E**) swarming and (**F**) swimming motility assays, a specific medium was used as described in Methods. For the analysis of (**G**) biofilm production, a modification of the Calgary device was used. Values that are significantly different by an unpaired two-tail *t*-test are indicated by asterisks as follows: * *p* < 0.05; ** *p* < 0.01; *** *p* < 0.001; **** *p* < 0.0001.

On the one hand, the results (Figure 8) showed that the MexJK-overexpressing mutant, *ΔmexL*, presented a decrease in the production of most of these virulence determinants (pyocyanin, rhamnolipids, pyoverdine, and biofilm), as well as an increase in swarming and swimming motilities. These results are in concordance with a decreased *Pqs*-dependent QS response [73]. On the other hand, the *ΔmexK* mutant maintained similar levels in the activity of these virulence determinants with respect to those obtained in the wild-type strain, except for a slight decrease in both swarming and swimming motilities (Figure 8E,F). No significant differences between the three strains in elastase production were detected (Figure 8B). With respect to pyoverdine production (Figure 8D), a siderophore implicated in iron uptake [74,75], the overexpression of MexJK produced a strong decrease in the production of this virulence factor, but the loss of MexJK functionality led to the opposite effect, since a significant increase in the production of this compound was observed in the *ΔmexK* mutant. This fact suggests that MexJK could be more directly implicated in the QS-dependent modulation of iron homeostasis, which interestingly is mainly carried out by the *Pqs*-system.

## 3. Materials and Methods

### 3.1. Bacterial Strains, Plasmids, and Primers

The *P. aeruginosa* and *Escherichia coli* strains and the plasmids used in this work are shown in Table 2. The primers used are listed in Table 3.

**Table 2:** Bacterial strains and plasmids used in this work.

Bacterial Strains/Plasmids	Description	Reference/Origin
*Pseudomonas aeruginosa*		
*P. aeruginosa* PAO1-Lausanne (PAO1)	Wild-type PAO1-L strain	Lab collection
*P. aeruginosa ΔmexL*	PAO1 strain which overproduces the MexJK efflux system by partial deletion of the *mexL* gene	Present work
*P. aeruginosa ΔmexK*	PAO1 strain with an inactive MexJK efflux system by partial deletion of the *mexK* gene	Present work
PAO1 CTX::*P_pqsA_-lux::pqsA* (PqsR-based biosensor)	Biosensor strain used for detecting AQs produced by *P. aeruginosa* strains	[72]
*P. aeruginosa* PAO1-L miniCTX-*lux*	PAO1 strain with the construction miniCTX-*lux* inserted in a neutral site of the chromosome	Present work
*P. aeruginosa ΔmexL* miniCTX-*lux*	*ΔmexL* strain with the construction miniCTX-*lux* inserted in a neutral site of the chromosome	Present work
*P. aeruginosa ΔmexK* miniCTX-*lux*	*ΔmexK* strain with the construction miniCTX-*lux* inserted in a neutral site of the chromosome	Present work
*P. aeruginosa* PAO1-L CTX-*lux*::P*lasI*	PAO1 strain with the construction miniCTX::P*_lasI_*-*lux* inserted in a neutral site of the chromosome	Present work
*P. aeruginosa ΔmexL* CTX-*lux*::P*lasI*	*ΔmexL* strain with the construction miniCTX::P*_lasI_*-*lux* inserted in a neutral site of the chromosome	Present work
*P. aeruginosa ΔmexK* CTX-*lux*::P*lasI*	*ΔmexK* strain with the construction miniCTX::P*_lasI_*-*lux* inserted in a neutral site of the chromosome	Present work
*P. aeruginosa* PAO1-L CTX-*lux*::P*rhlI*	PAO1 strain with the construction miniCTX::P*_rhlI_*-*lux* inserted in a neutral site of the chromosome	Present work
*P. aeruginosa ΔmexL* CTX*-lux::*P*rhlI*	*ΔmexL* strain with the construction miniCTX::P*_rhlI_*-*lux* inserted in a neutral site of the chromosome	Present work
*P. aeruginosa ΔmexK* CTX-*lux*::P*rhlI*	*ΔmexK* strain with the construction miniCTX::P*_rhlI_*-*lux* inserted in a neutral site of the chromosome	Present work
*P. aeruginosa* PAO1-L CTX-*lux*::P*pqsA*	PAO1 strain with the construction miniCTX::P*_pqsA_-lux* inserted in a neutral site of the chromosome	Present work
*P. aeruginosa ΔmexL* CTX*-lux::*P*pqsA*	*ΔmexL* strain with the construction miniCTX::P*_pqsA_-lux* inserted in a neutral site of the chromosome	Present work
*P. aeruginosa ΔmexK* CTX-*lux*::P*pqsA*	*ΔmexK* strain with the construction miniCTX::P*_pqsA_-lux* inserted in a neutral site of the chromosome	Present work
***Escherichia coli***		
S17 λ*pir*	Strain used for transferring plasmids in conjugation assays: F^−^ thi pro hsdR hsdM^+^ recA RP42-Tc::Mu-Km::Tn7	[76]
S17 λ*pir* (miniCTX-*lux*)	S17 λ*pir* strain used for transferring the plasmid Mini-CTX-*lux* in conjugation assays	[72]
S17 λ*pir* (miniCTX::P*_lasI_*-*lux*)	S17 λ*pir* strain used for transferring the plasmid miniCTX::P*_lasI_*-*lux* in conjugation assays	Borrowed from Miguel Cámara
S17 λ*pir* (miniCTX::P*_rhlI_*-*lux*)	S17 λ*pir* strain used for transferring the plasmid miniCTX::P*_rhlI_*-*lux* in conjugation assays	[24]
S17 λ*pir* (miniCTX::P*_pqsA_-lux*)	S17 λ*pir* strain used for transferring the plasmid miniCTX::P*_pqsA_-lux* in conjugation assays	[72]
S17 λ*pir* (pEX18-Ap-*ΔmexL*)	S17 λ*pir* strain used for transferring the plasmid pEX18-Ap_*ΔmexL* in conjugation assays	Present work
S17 λ*pir* (pEX18-Ap-*ΔmexK*)	S17 λ*pir* strain used for transferring the plasmid pEX18-Ap_*ΔmexK* in conjugation assays	Present work
One Shot OmniMax™ 2 T1	Host strain used for the maintenance of cloning plasmids: F′ {*pro*AB *lac*I^q^ *lac*Z*Δ*M15 *Tn*10(Tet^R^) *Δ*(*ccd*AB) *mcr*A, *Δ*(*mrr*,*hsd*RMS-*mcr*BC) ɸ80(*lac*Z)*Δ*M15 *Δ*(*lac*ZYA-*arg*F)U169 *end*A1 recA1 *sup*E44 *thi*-1 *gyr*A96 *rel*A1 *ton*A *pan*D	Invitrogen
OmniMax™ (pEX18-Ap-*ΔmexL*)	One Shot OmniMax™ 2 T1 harboring the plasmid pEX18-Ap_*ΔmexL*	Present work
OmniMax™ (pEX18-Ap-*ΔmexK*)	One Shot OmniMax™ 2 T1 harboring the plasmid pEX18-Ap_*ΔmexK*	Present work
JM109-pSB1142 (LasR-based biosensor)	Biosensor strain used for detecting 3-oxo-C12-HSL produced by *P. aeruginosa* strains	[77]
JM109-pSB536 (RhlR-based biosensor)	Biosensor strain used for detecting C4-HSL produced by *P. aeruginosa* strains	[78]
Plasmids		
miniCTX-*lux*	Reporter plasmid with the *luxCDABE* operon. Tc^R^	[79]
miniCTX::P*_lasI_*-*lux*	Reporter plasmid with the l*uxCDABE* operon under the control of P*lasI* promoter. Tc^R^	Borrowed from Miguel Cámara
miniCTX::P*_rhlI_*-*lux*	Reporter plasmid with the l*uxCDABE* operon under the control of P*rhlI* promoter. Tc^R^	[24]
miniCTX::P*_pqsA_-lux*	Reporter plasmid with the l*uxCDABE* operon under the control of P*pqsA* promoter. Tc^R^	[72]
pGEM-T Easy	Commercial plasmid “pGEM-T Easy Vector” used for cloning optimization of PCR products. Amp^R^	Promega
pGEM-T Easy-*ΔmexL*	Commercial plasmid “pGEM-T Easy Vector” (Promega) used for cloning optimization of the flanking region of *mexL* gene. Amp^R^	Present work
pGEM-T Easy-*ΔmexK*	Commercial plasmid “pGEM-T Easy Vector” used for cloning optimization of the flanking region of *mexK* gene. Amp^R^	Present work
pEX18-Ap	Plasmid with conjugative properties used for deleting genes in *P. aeruginosa* by homologous recombination. Amp^R^	[80]
pEX18-Ap-*ΔmexL*	Plasmid with conjugative properties containing “Up and down *mexL* insert” used for deleting *mexL* gene in *P. aeruginosa* by homologous recombination. Amp^R^	Present work
pEX18-Ap-*ΔmexK*	Plasmid with conjugative properties containing “Up and down *mexK* insert” used for deleting *mexK* gene in *P. aeruginosa* by homologous recombination. Amp^R^	Present work

### 3.2. Growth Media, Culture Conditions, and Antibiotic Susceptibility Testing

Unless other conditions are specified, routine experiments with *P. aeruginosa* were carried out at 37 °C and 250 rpm in 100 mL glass flasks containing 25 mL of LB (Lysogeny Broth, Pronadisa Condalab, Torrejón de Ardoz, Spain), which were inoculated at the beginning of the experiments at OD_600_ = 0.01. Antibiotic susceptibility assays were performed using MIC test strips (Liofilchem, Roseto degli Abruzzi TE, Italia) on Mueller-Hinton (MH) agar (Pronadisa Condalab, Torrejón de Ardoz, Spain) plates. Overnight bacterial cultures were diluted to an OD_600_ of 0.01 in sterile 0.85% NaCl. Later, 120 μL of this suspension was placed and spread in Petri dishes containing 20 mL of MH agar. Afterwards, MIC test strips were deposited on each MH agar plate. Plates were incubated for 18–20 h at 37 °C to finally measure the growth inhibition halo generated by the corresponding antibiotics.

Growth in LB and in the presence of 64 μg/mL erythromycin, 0.06 μg/mL ciprofloxacin, or 109 μg/mL triclosan was measured with a Spark 10M plate reader (Tecan, Männedorf, Switzerland) at OD_600_ in flat-bottomed transparent 96-well plates (Nunc MicroWell; Thermo Fisher; Waltham, MA, USA). The stock solutions of each compound were diluted in LB medium to obtain the required concentrations. Then, 10 µL of cell culture was inoculated in 140 µL of medium in each well, to a final OD_600_ of 0.01. The plates were incubated at 37 °C with 10 s of shaking every 10 min for 24 h.

For the swarming assay, the medium was composed of a mix of 0.5% Casamino Acids, 0.5% Bacto agar, 0.5% filtered glucose, 3.3 mM K_2_HPO_4_, and 3 mM MgSO_4_. The swimming motility was determined on LB agar (0.3%) plates. Afterwards, 25 mL of each medium was poured into Petri dishes.

### 3.3. Generation of ΔmexL and ΔmexK Mutant Strains

The generation of *ΔmexL* and *ΔmexK* strains was carried out by double homologous recombination as stated [80]. Two fragments of about 500 bp flanking the areas to be deleted were amplified by PCR using the primers listed in Table 3. These fragments were purified from agarose gel electrophoresis and used for nested overlapping PCRs, thus generating fragments “Up and Down”, which were cloned into pGEM-t Easy vector and introduced into *E. coli* One Shot OmniMax™ 2 T1 competent cells (Thermo Fisher, Waltham, MA, USA) by transformation (LB plate with ampicillin 100 µg/mL was used to select transformant colonies). Once the absence of SNPs, potentially introduced by PCR, was confirmed by Sanger sequencing, the pGEM-t-based plasmids and a pEX18-Ap empty vector were isolated, purified, and digested with EcoRI and BamHI (New England Biolabs). The linearized pEX18-Ap empty vector and both flanking regions of *mexL* and *mexK* genes were purified and independently ligated using T4 DNA ligase (New England Biolabs). The resulting constructions, pEX18-Ap-*ΔmexL* and pEX18-Ap-*ΔmexK*, were incorporated into *E. coli* S17-1 λ*pir* by transformation (LB plate with ampicillin 100 µg/mL was used to select transformant colonies). Next, the conjugation of the wild-type strain (PAO1) with the strains carrying the pEX18-Ap vectors was carried out, and a double recombination in two steps was forced in petri dishes containing (i) Pseudomonas Isolation Agar (PIA) containing carbenicillin 200 µg/mL (first recombination: integration of the plasmid in the homologous-recombination region of the chromosome) and (ii) PIA supplemented with sucrose 10% (second recombination: split of the nonhomologous region of pEX18-Ap vectors based on its toxicity in the presence of sucrose) to obtain *ΔmexL* and *ΔmexK* mutants. Finally, the resulting colonies were checked by PCR using the paired primers mexL_Fwd_Check/mexL_Rev_Check and mexK_Fwd_Check/mexK_Rev_Check to confirm the deletion of the respective genes.

### 3.4. RNA Preparation and RT-qPCR

Overnight cultures of three biological replicates of each strain of *P. aeruginosa* were washed and diluted in LB broth to an OD_600_ of 0.01. They were grown to exponential phase (OD_600_ = 0.6) and then diluted again to an OD_600_ of 0.01. The cultures were incubated until they reached the exponential (OD_600_ = 0.6) or early stationary phase of growth (OD_600_ = 2.5). Afterwards, 10 mL of each culture was centrifuged at 7000× *g* for 20 min. The cell pellets were resuspended in 570 μL of TE buffer with 20 μL of lysozyme (20 mg/mL), and the mixtures were incubated at room temperature for 10 min. Then, 2100 μL of RLT buffer (1% β-mercaptoethanol) from the RNeasy mini kit (QIAGEN, Hilden, Germany) was added, and the samples were sonicated in two 30 s cycles (constant frequency, 0.45 Hz). Subsequently, 1410 μL of 100% ethanol (Merck, Darmstadt, Germany) was added and mixed; the extraction continued following the instructions of the manufacturer. Finally, 10 μg of ARN was retrotranscripted using the commercial kit QuantiTect Reverse Transcription kit (QIAGEN, Hilden, Germany).

Triplicated RT-qPCR assays were carried out on 96-well plates with 50 ng RNA in a final volume of 25 μL, using an Applied Biosystems^®^ 7500 Real-Time PCR thermal cycler. The values were normalized to the values of the reference housekeeping gene, *rpsL*. The relative expression of each gene was calculated based on the 2^−*ΔΔ*Ct^ method [81].

### 3.5. Detection of QS Molecules

The AHLs-type QS molecules were extracted from cultures in late exponential phase at an OD_600_ of 1.7, while those of the AQs type were extracted from cultures in early stationary phase at an OD_600_ of 2.5, following in both cases the methodology previously described, from each one of the three biological replicates analyzed in the respective strain [24].

For AQs detection by TLC, the silica gel 60 F254 (Merck, Darmstadt, Germany) plates were activated by dipping for 30 min in a 5% (*p*/*v*) KH_2_PO_4_ solution and dried in an oven at 80 °C for 90 min. The samples were resuspended in 100 μL of HPLC-grade methanol, of which 20 μL was loaded onto the silica gel sheet. As a positive control, 2 μL of 10 mM synthetic PQS and HHQ and 4 μL of the mixture of both were loaded too. The mobile phase used for the chromatography was a mixture of dichloromethane:methanol (95:5). The detection of PQS and HHQ was carried out by culturing on the silica gel sheet the PqsR-based biosensor (Table 2), which emits light in the presence of PQS and HHQ in a concentration-dependent way. For that, an overnight culture of PqsR-based biosensor was diluted to 1/100 in soft top agar medium (0.65% agar, 1% peptone, 0.5% NaCl), which was then poured on the TLC plate and incubated for 6 h at 37 °C.

For AHLs detection, the supernatants were resuspended in 1 mL and cell extracts in 200 μL of methanol (grade HPLC). The TLC plates used for the detection of 3-oxo-C12-HSL and C4-HSL were silica gel 60 F254 (Merck) and silica gel 60 RP-18 F254, respectively. For 3-oxo-C12-HSL detection, 10 μL of cell extract and 1 μL of supernatant were loaded, whereas for C4-HSL analysis, 40 μL of the cell extract and 20 μL of the supernatants were loaded. As a positive control, 4 μL of synthetic C4-HSL or 3-oxo-C12-HSL 10 mM was loaded. The chromatography was carried out with a mixture of methanol:water (60:40) as mobile phase, letting the samples run for approximately 90 min. Afterwards, the AHLs were detected by culturing on top of the said sheet the corresponding biosensor strains using 0.75% LB agar medium and incubating for 18 h. For the detection of 3-oxo-C12-HSL, the LasR-based biosensor was used, while for the detection of C4-HSL, we used the RhlR-based biosensor (Table 2). In both cases, the bioluminescence associated to AQs and AHLs detection was quantified by densitometry analysis of the spots using the image processing software ImageJ. Each detection was performed three times.

### 3.6. Analysis of the Activation Kinetics of the Promoters PlasI, PrhlI, and PpqsA throughout the Growth Cycle

For the promoters’ expression analysis, we inserted into a specific and neutral site of the chromosome of PAO1, *ΔmexL*, and *ΔmexK* strains each one of the miniCTX-*lux* derived plasmids (miniCTX-*lux*, miniCTX::P*_lasI_*-*lux*, miniCTX::P*_rhlI_*-*lux*, and miniCTX::P*_pqsA_*-*lux*) following the protocol described by Hoang et al. [82] with some modifications [24]. The recipient *P. aeruginosa* strains and the donors *E. coli* S17-1λ*pir* harboring the corresponding plasmids were grown for 16 h at 42 °C in 50 mL flasks containing 10 mL of LB. Subsequently, 1 mL aliquots from donor and receptor strains were mixed and concentrated and then spotted on LB agar plates and incubated for 8 h at 37 °C. After this incubation, cells were recovered, resuspended in 1 mL of 0.85% NaCl, and different dilutions were seeded in Petri dishes containing PIA with 100 µg/mL tetracycline, thus forcing the selection of recipient *P. aeruginosa* strains with the respective miniCTX-*lux* derived plasmids integrated into the chromosome. We then selected the luminescent colonies, and the correct insertion of the construct was confirmed by PCR using the CTX-Fw and CTX-Rev primers (Table 3).

To analyze the time-course promoter activity, the protocol established by Laborda, P. et al. was performed [15]. Three biological replicates of overnight cultures were inoculated into wells of flat-bottomed transparent plates at an OD_600_ of 0.01. The measure of luminescence was taken every 10 min for a period of at least 20 h using a TECAN infinite 200 multiplate reader. The ratio between bioluminescence (RLU) and absorbance (OD_600_) for each strain and time was calculated, represented in a graphic, and the area under the curves were quantified, obtaining a single numeric value for each strain and promoter construction analyzed. Finally, the values obtained for each strain (PAO1, *ΔmexL*, and *ΔmexK* strains with CTX::P*_lasI_*-*lux*, CTX::P*_rhlI_*-*lux*, and CTX::P*_pqsA_*-*lux* constructions) were normalized with respect to those obtained in their corresponding control strain (PAO1, *ΔmexL*, and *ΔmexK* strains with miniCTX-*lux* construction). Each experiment was performed three times with five technical replicates.

### 3.7. Analysis of the Production of QS-Regulated Virulence Factors

In all cases, the preinocula were grown overnight in 50 mL flasks containing 10 mL of LB medium, and the experiments were carried out in triplicate. For the production of pyocyanin, pyoverdine, elastase, and rhamnolipids, the preinocula were washed with fresh LB medium, diluted to an OD_600_ of 0.01, and incubated for 20 h at 37 °C with constant shaking before measuring the production of each virulence factor. All experiments were performed three times with three technical replicates.

Pyocyanin production was determined following the method described by Essar et al. [83] with some modifications. Culture supernatants of 7.5 mL were mixed with 4.5 mL of chloroform. The samples were centrifuged, and the lower phase containing pyocyanin was mixed with 1.5 mL of HCl at 0.2 N. After a new centrifugation, 900 μL of the upper phase was added to a spectrophotometry cuvette and the absorbance at a wavelength of 520 nm was measured. The concentration of pyocyanin was calculated based on its molar extinction coefficient.

To measure elastase secretion in *P. aeruginosa* strains, we followed the methods established by Kessler and Safrin [84] with some modifications. Briefly, 100 μL of the free supernatant was mixed with 1 mL of an elastin–Congo Red solution (5 mg/mL) previously suspended in 100 mM Tris–HCl and 1 mM CaCl at 7.5 pH. The reactions were incubated for 2 h at 37 °C under constant shaking. The samples were centrifuged at 7000 rpm for 10 min, and the absorbance of the supernatant was measured at a wavelength of 495 nm.

The procedure used for the extraction and detection of mono- and di-rhamnolipids by TLC was carried out following the indications of Wittgens et al. [85] with minor modifications. Cell-free supernatant (5 mL) was mixed with the same volume of ethyl acetate acidified at a ratio 1:10.000, followed by a centrifugation at 7000× *g* for 20 min to separate both phases. Subsequently, 10 mL of the upper phase corresponding to ethyl acetate was extracted, and the process was repeated again with another 10 mL of acidified ethyl acetate. The recollected ethyl acetate, in which the rhamnolipids are dissolved, was concentrated by evaporation using a rotary evaporator and was resuspended in 40 µL of ethanol. The amount of mono- and di-rhamnolipids present in 5 µL of these samples was analyzed by TLC.

For swarming motility test, the preinocula were washed with 0.85% NaCl and diluted to an OD_600_ of 1.0, as previously stated [23]. Subsequently, a 5 μL drop of this cell suspension was placed on the center of Petri dishes containing 25 mL of swarming medium and grown for 16 h at 37 °C. The swimming motility of all the tested strains was determined on LB agar (0.3%) plates. An overnight culture from each strain was diluted to a final OD_600_ of 2, and 5 µL was spotted on the surface of the swimming plates and incubated for 24 h at 30 °C. For the swarming and swimming motility assays, pictures were taken and the growth zone was measured with ImageJ software.

Pyoverdine production was measured using the method described by Hoegy et al. [86]. Overnight cultures were diluted in a 1:10 ratio with 50 mM Tris–HCl at pH 8. The samples were placed in a 96-well plate, and the fluorescence emitted at 447 nm was measure after exciting the sample to a 400 nm wavelength using a TECAN Spark multiplate reader.

Biofilm formation was analyzed as previously described [87]. One hundred and fifty microliters of an overnight bacterial culture wwas added to a p96 plate with 96 conical structures (NUNC-IMMUNO PLATE), covered with a lid on which the biofilm adhered to. They were grown overnight at 37 °C without shaking. The adhered biofilm was then stained with 25 μL of 0.1% crystal violet, and, after 5 min, the excess of dye was washed five times with distilled water. Finally, the biofilm from the walls was removed with 0.25% Triton X-100, and biofilm quantification was performed by measuring OD_570_ nm.

## 4. Conclusions

MexJK-overexpressing mutants have been previously isolated from infected patients and, together with *pqsR* and *lasR* mutants, from in vitro evolution of biofilm populations [65,68,69], which supports that this efflux pump should be of relevance for the behavior of *P. aeruginosa* under infective conditions. However, despite previous claims [58,59,60], our results suggest that the contribution of this efflux system to antibiotic resistance in bacteria with a wild-type genomic background, harboring all intrinsic efflux-pumps-encoding genes, is minor, if any. These findings support that MexJK has an unknown role in *P. aeruginosa* physiology, beyond antibiotic resistance. Further, it has been previously shown that calcium, an important signal molecule in eukaryotic cells, triggers the expression of MexJK, and this efflux system is of relevance for calcium homeostasis and calcium-induced plant infectivity in *P. aeruginosa* [88]. Additionally, it has been shown that *mexJK* expression is regulated by QS, which suggests that this efflux system may modulate the QS response, as has been observed for other RND efflux systems (Figure 1) in this bacterium [20,23,24,25,26,47,48,50]. Our results support this last hypothesis, since we found that the overexpression of MexJK leads to an impaired production of QSSMs, resulting in a defective QS response and changes in the expression level and the activity of several QS-regulated virulence factors, mainly those depending on the *Pqs* system. Among them, the strong impact of MexJK activity in pyoverdine production—impaired when *mexJK* is overexpressed and higher when this efflux pump is absent—suggests that this efflux system could participate in *P. aeruginosa* iron homeostasis driven by pyoverdine production. These results, together with the above-mentioned works, support the concept that, in contrast to previous claims [58,59,60], the main role of MexJK is related to virulence and in vivo host interactions rather than to antibiotic resistance.

## Figures and Tables

**Table 1:** MICs of different antibiotics for PAO1, *ΔmexL*, and *ΔmexK* strains.

Antibiotic	Strain			Antibiotic	Strain		
	**PAO1**	***ΔmexL***	***ΔmexK***		**PAO1**	***ΔmexL***	***ΔmexK***
Amikacin	3	1.5	1.5	Cefuroxime	>256	>256	>256
Gentamicin	1	1	1.5	Cephalothin	>256	>256	>256
Kanamycin	24	32	32	Ampicillin	>256	>256	>256
Streptomycin	24	16	16	Aztreonam	1.5	1.5	1.5
Tobramycin	1	0.75	0.75	Imipenem	6	4	4
Tetracycline	12	8	6	Ertapenem	16	16	16
Tigecycline	12	8	12	Meropenem	1	1	1
Nalidixic acid	128	96	96	Piperacillin	3	2	3
Ciprofloxacin	0.064	0.047	0.064	Oxacillin	>256	>256	>256
Gatifloxacin	0.25	0.25	0.19	Colistin	2	2	2
Levofloxacin	0.25	0.25	0.25	Polymyxin B	1	1	1.5
Moxifloxacin	1.5	1.5	2	Chloramphenicol	64	64	64
Norfloxacin	0.38	0.38	0.38	Erythromycin	>256	>256	>256
Ofloxacin	0.75	0.75	0.75	Fosfomycin	256	256	256
Cefepime	0.75	0.75	0.75	Rifampicin	12	12	12
Cefoxitin	>256	>256	>256	Trimethoprim-Sulfamethoxazole	1.5	2	1.5
Cefotaxime	12	8	8	Ceftazidime–Avibactam	0.75	0.75	0.75
Ceftazidime	0.5	0.5	0.5				

**Table 3:** Primers used in the present work.

Name	Sequence	Description
CTX-Fwd	5′-GTCATGCTCTTCTCTAATGCGTG-3′	Check the insertion of mini-CTX-lux in the chromosome of *P. aeruginosa* strains
CTX-Rev	5′-GCGTAATACGACTCACTATAGGGC-3′	
*rplU* Fwd	5′-CGCAGTGATTGTTACCGGTG-3′	Check DNA contamination of RNA samples
*rplU* Rev	5′-AGGCCTGAATGCCGGTGATC-3′	
EcoRI_mexL_Fwd	5′- CCCGAATTCCTGGGAATGGCTGACCAGGT-3′	Amplification of flanking area “Up” around *mexL* gene
mexL_int_Rev	5′-CGTCGCGCCTGAGCTGCCGCGCTTATACAATTGAAA-3′	
mexL_int_Fwd	5′-TTTCAATTGTATAAGCGCGGCAGCTCAGGCGCGACG-3′	Amplification of flanking area “Down” around *mexL* gene
BamHI_mexL_Rev	5′-CCCGGATCCTGCTCGCGCGGCTACGC-3′	
mexL_Fwd_Check	5′-CGAGGAACAGGGAGGAAAAC-3′	Check *mexL* deletion
mexL_Rev_Check	5′-GGCGCCTACTTCCCCTTC-3′	
EcoRI_mexK_Fw	5′-CCCGAATTCCGAGGTGCTGATCGGCCTGC-3′	Amplification of flanking area “Up” around *mexK* gene
mexK_int_Rev	5′-GTCCCTTCTCCCGTCAGGGCGACTACTCCTTGGCCG-3′	
mexK_int_Fwd	5′-CGGCCAAGGAGTAGTCGCCCTGACGGGAGAAGGGAC-3′	Amplification of flanking area “Down” around *mexK* gene
BamHI_mexK_Rev	5′-CCCGGATCCAGCTGATGAAGCAGTTCGGC-3′	
mexK_Fwd_Check	5′-GGTGCTCGAAGGCCTGAA-3′	Check *mexK* deletion
mexK_Rev_Check	5′-AACGTCGAGGGCTATGTCAC-3′	
*mexK* Fwd	5′-GGTGCTCGAAGGCCTGAA-3′	RT-qPCR
*mexK* Rev	5′-AACGTCGAGGGCTATGTCAC-3′	
*hcnB* Fwd	5′-GAACGCCGAGAATCCCATCT-3′	RT-qPCR
*hcnB* Rev	5′-CATCGCCGGGCTGAAGAT-3′	
*lasA* Fwd	5′-ATGGACCAGATCCAGGTGAG-3′	RT-qPCR
*lasA* Rev	5′-CGTTGTCGTAGTTGCTGGTG-3′	
*lasB* Fwd	5′-ATCGGCAAGTACACCTACGG-3′	RT-qPCR
*lasB* Rev	5′-ACCAGTCCCGGTACAGTTTG-3′	
*lasI* Fwd	5′-CTACAGCCTGCAGAACGACA-3′	RT-qPCR
*lasI* Rev	5′-ATCTGGGTCTTGGCATTGAG-3′	
*mexB* Fwd	5′-TGAACAGCGTGTTCGAACTGG-3′	RT-qPCR
*mexB* Rev	5′-CACTTCGACATTACGAATCCC-3′	
*mexC* Fwd	5′-GAAGCGCTTCGAGGAGGG-3′	RT-qPCR
*mexC* Rev	5′-CAGCCAGCAGGACTTCGATA-3′	
*mexE* Fwd	5′-GGACTTCCTCGACAACCAGG-3′	RT-qPCR
*mexE* Rev	5′-AGAAGTTCGTGCTGGTCCTG-3′	
*mexG* Fwd	5′-GGCGAAGCTGTTCGACTATC-3′	RT-qPCR
*mexG* Rev	5′-AGAAGGTGTGGACGATGAGG-3′	
*mexX* Fwd	5′-GTCGCCCTATTCCTGCTGG-3′	RT-qPCR
*mexX* Rev	5′-GTCACCCGTCGCCTGTAC-3′	
*opmH* Fwd	5′-GTGCGCGACTACAACAACAG-3′	RT-qPCR
*opmH* Rev	5′-GCCGAGCAGTTACAGAGCAA-3′	
*oprM* Fwd	5′-TCAACCTGCCGATCTTCACC-3′	RT-qPCR
*oprM* Rev	5′-GCGACGAGTACTACCAGCTC-3′	
*phnB* Fwd	5′-CACTCGCTGGTGGTCAGTC-3′	RT-qPCR
*phnB* Rev	5′-AGAGTAGAGCGTTCTCCAGCA-3′	
*phzB1* Fwd	5′-AACGAACTTCGCGAAAAGAA-3′	RT-qPCR
*phzB1* Rev	5′-TTTGTCTTTGCCACGAATGA-3′	
*phzB2* Fwd	5′-GCGAGACGGTGGTCAAGTAT-3′	RT-qPCR
*phzB2* Rev	5′-AATCCGGGAAGCATTTCAG-3′	
phzS Fwd	5′-CAAGTCGCTGGTGAACTGG-3′	RT-qPCR
phzS Rev	5′-CGGGTACTGCAGGATCAACT-3′	
*pqsA* Fwd	5′-CAATACACCTCGGGTTCCAC-3′	RT-qPCR
*pqsA* Rev	5′-TGAACCAGGGAAAGAACAGG-3′	
*pqsE* Fwd	5′-TGGTGTTCGACGACATGGAG-3′	RT-qPCR
*pqsE* Rev	5′-AATCCCTCGACGAACTGAGC-3′	
*pqsH* Fwd	5′-ATGTCTACGCGACCCTGAAG-3′	RT-qPCR
*pqsH* Rev	5′-AACTCCTCGAGGTCGTTGTG-3′	
*rhlA* Fwd	5′-CGAGGTCAATCACCTGGTCT-3′	RT-qPCR
*rhlA* Rev	5′-GACGGTCTCGTTGAGCAGAT-3′	
*rhlI* Fwd	5′-CTCTCTGAATCGCTGGAAGG-3′	RT-qPCR
*rhlI* Rev	5′-GACGTCCTTGAGCAGGTAGG-3′	
*rpsL* Fwd	5′-GCAAGCGCATGGTCGACAAGA-3′	RT-qPCR (housekeeping)
*rpsL* Rev	5′-CGCTGTGCTCTTGCAGGTTGTGA-3′	

## Data Availability

All data used in the work have been included in the manuscript.
